# A Bioinspired Soft Manipulator Based on Wave Spring Structure: Design and Control

**DOI:** 10.3390/biomimetics11090629

**Published:** 2026-09-03

**Authors:** Dongbao Sui, Zongwei Zhang, Tianshuo Wang, Sikai Zhao, Yu Zhang, Xinzui Wang

**Affiliations:** 1 Ji Hua Laboratory, Foshan 528200, China; suidb@jihualab.ac.cn (D.S.); zhangzw@jihualab.ac.cn (Z.Z.); wangts@jihualab.ac.cn (T.W.); wangxz@jihualab.ac.cn (X.W.); 2 State Key Laboratory of Robotics and Systems, Harbin Institute of Technology, Harbin 150001, China; 3 Heilongjiang Provincial Key Laboratory of Complex Intelligent System and Integration, Harbin University of Science and Technology, Harbin 150080, China; yuzhang@hrbust.edu.cn

**Keywords:** continuum robot, wave spring structure, compliant control, iterative learning control

## Abstract

Architectured soft structures have unlocked new possibilities for designing continuum manipulators with tailored mechanical performance. This work introduces a bioinspired soft manipulator based on modular wave spring units, leveraging the high elasticity of wave spring structures to enable compliant deformation and axial extensibility of the soft manipulator. To achieve precise control of the soft manipulator, a neural network-based inverse kinematics framework is developed to establish an efficient mapping from desired end poses to tendon actuation lengths. An iterative learning control strategy is further integrated to compensate for material hysteresis, friction, and external disturbances. A prototype is fabricated using flexible 3D-printable material (TPU), and comprehensive experiments are conducted to validate extensible performance, point positioning accuracy, trajectory-tracking accuracy, and compliant interaction capability. The results demonstrate that the proposed wave spring-based soft manipulator achieves a high extension ratio and reliable control precision.

## 1. Introduction

Soft continuum robots, inspired by biological muscular hydrostats such as elephant trunks, octopus tentacles, and snake bodies, have emerged as a transformative research area in modern robotics [1,2,3]. Unlike traditional rigid robots constructed with discrete joints and rigid links, continuum robots feature compliant, continuous structures capable of infinite degrees of freedom and smooth, organic motion [4,5]. This inherent compliance enables safe physical interaction with humans and delicate environments, making them highly promising for applications including minimally invasive surgery, industrial inspection, search-and-rescue operations, and collaborative manipulation [6,7]. Despite remarkable progress over the past decade, the development of high-performance soft continuum robots still faces two fundamental bottlenecks: the design of architectured structures that simultaneously achieve large deformation, structural stability, and tunable mechanical properties; and the realization of precise, robust control under strong nonlinearity, hysteresis, and model uncertainty [8,9].

Architectured soft structures, which tailor mechanical behavior through geometric topology rather than material composition alone, have become a key enabler for advancing continuum robot design [10,11]. By precisely designing spatial geometry, these structures can decouple conflicting mechanical requirements, such as high compliance and structural rigidity, or large deformation and load-bearing capacity. Among representative architectured designs, trimmed helicoids have recently demonstrated exceptional performance for soft manipulators [9]. This structure consists of helicoidal surfaces radially trimmed along the central axis, enabling independent regulation of axial and bending stiffness through geometric parameters such as helical angle and trimming width. However, the fabrication of trimmed helicoids requires complex geometric optimization, and their axial extensibility is limited, restricting applications requiring large-range extensible motion.

Spring-based structures, including helical springs and wave springs, represent another important category of architectured soft robots [12,13]. Wave springs, composed of periodic wavy geometries, exhibit unique advantages: high elasticity, lightweight, modularity, and excellent axial extensibility [12]. Unlike conventional helical springs, wave springs generate large linear deformation under small axial loads while maintaining structural stability against buckling [13]. Recent studies have validated wave springs as soft actuator skeletons and continuum robot segments, demonstrating their potential for large-range extension/contraction and omnidirectional bending [14,15]. However, existing wave spring designs focus primarily on single-module performance, lacking systematic integration into multi-degree-of-freedom (DoF) serial manipulators. Moreover, the mechanical behavior of serial wave spring assemblies remains underexplored, limiting their application in high-precision manipulation tasks.

Beyond trimmed helicoids and wave springs, other mainstream soft robot structures include pneumatic networks, origami-inspired architectures, and auxetic cylinders [16,17,18]. Pneumatic soft robots use air pressure to drive deformation, offering simple actuation and large workspace but suffering from low precision and poor structural stability under large loads [18]. Origami-inspired structures leverage folding mechanics to achieve programmable deformation, but their deformation range is limited, and fabrication complexity is high [16]. Auxetic structures exhibit negative Poisson’s ratio, enabling unique deformation modes, but their mechanical strength is generally low [17]. Despite their respective advantages, these structures struggle to simultaneously satisfy large axial extensibility, stable bending deformation, and easy modular assembly, highlighting the need for novel architectured designs [19].

In parallel with structural innovation, precision control of soft continuum robots remains a critical challenge, primarily due to strong nonlinearity, material hysteresis, and complex deformation kinematics [20,21]. Current control methods are broadly divided into model-based and model-free approaches, each with distinct advantages and limitations. Model-based control relies on analytical kinematic or dynamic models to establish mappings between actuation inputs and end outputs [20]. The most widely used model is the piecewise constant-curvature (PCC) model, which approximates the robot’s shape as a series of constant-curvature segments. While the PCC model is simple and computationally efficient, it fails to accurately describe large deformation, material hysteresis, and external disturbances, leading to significant control errors [22]. More advanced models, such as the Cosserat rod theory, incorporate distributed forces and moments, improving accuracy but increasing computational complexity and requiring precise system parameters [21]. These limitations restrict the practical application of model-based control in real-world scenarios.

Model-free control methods, by contrast, bypass complex analytical modeling and directly learn control laws from input–output data, making them highly adaptable to nonlinear systems [23,24]. Neural networks, in particular, have been widely adopted for inverse kinematics approximation, effectively mapping desired end poses to actuation commands without requiring explicit kinematic equations [23]. However, open-loop neural network control is vulnerable to model drift and external disturbances, limiting long-term tracking accuracy [25]. To address this, feedback control has emerged as a promising solution, compensating for real-time errors through closed-loop adjustment and significantly improving trajectory precision [26]. Despite these advances, most existing model-free control frameworks are validated on low-DoF or small-scale soft robots, and their applicability to serial wave spring manipulators with large deformation and high elasticity remains underexplored.

Bioinspired design principles provide critical insights for addressing the above challenges, as nature has evolved remarkable soft appendages with both large deformation and precise motion capabilities [27]. Elephant trunks and octopus arms, for example, combine hierarchical compliant structures with distributed neuromuscular control, achieving both large-scale extension/contraction and delicate manipulation [6,7]. This biological inspiration has driven the development of modular continuum robots, where serially connected segments mimic the hierarchical flexibility of natural appendages [28]. Recent studies have advanced bioinspired continuum robot design and control: Jia et al. developed a modular hydraulic muscle-driven continuum robot with multi-DoF motion, validating bioinspired motion patterns and master–slave control performance [29]; Deng et al. designed a rigid–flexible coupled continuum robot inspired by octopus musculature, achieving high dexterity with simplified actuation [2]. These works highlight the potential of bioinspired modular design.

To address the above limitations, this work presents a bioinspired soft continuum manipulator based on modular wave spring units, leveraging the high elasticity of wave spring structures to enable compliant deformation and axial extensibility. The manipulator adopts a serial modular configuration, where interconnected wave spring units provide large-range extension/contraction and omnidirectional bending. To enable precise control of the nonlinear soft manipulator, a neural network-based inverse kinematics framework is developed to establish an efficient mapping from desired end poses to tendon actuation lengths. An iterative learning control strategy is further integrated to compensate for material hysteresis, friction, and external disturbances. A prototype is fabricated using flexible 3D-printable TPU material, and comprehensive experiments are conducted to validate extensible performance, point positioning accuracy, trajectory-tracking accuracy, and compliant interaction capability.

Most existing wave-spring soft-robot publications only investigate single-module wave spring units and verify their standalone deformation performance. Serial multi-DOF assembly design, gradient-stiffness implementation and complete system-level closed-loop control are rarely well studied. Our work realizes a two-segment serial manipulator with differentiated rib widths to achieve gradient stiffness mimicking the mechanical characteristics of elephant trunks. Moreover, we combine data-driven inverse kinematics with iterative-learning closed-loop compensation to mitigate large hysteresis and nonlinearity inherent to wave-spring soft structures, which has seldom been reported for wave-spring continuum manipulators.

The main contributions of this work are as follows: (1) A novel serial wave-spring soft manipulator design that leverages high elasticity for compliant deformation and axial extensibility; (2) a neural network-based inverse kinematics solver for efficient pose-to-actuation mapping; (3) an iterative learning control strategy to enhance precision and robustness; (4) experimental validation of the prototype’s performance in deformation, tracking, and interaction tasks. This work advances the design and control of architectured soft manipulators, providing a promising solution for bioinspired precision manipulation.

## 2. Design of the Bioinspired Soft Manipulator

**Figure 1:**
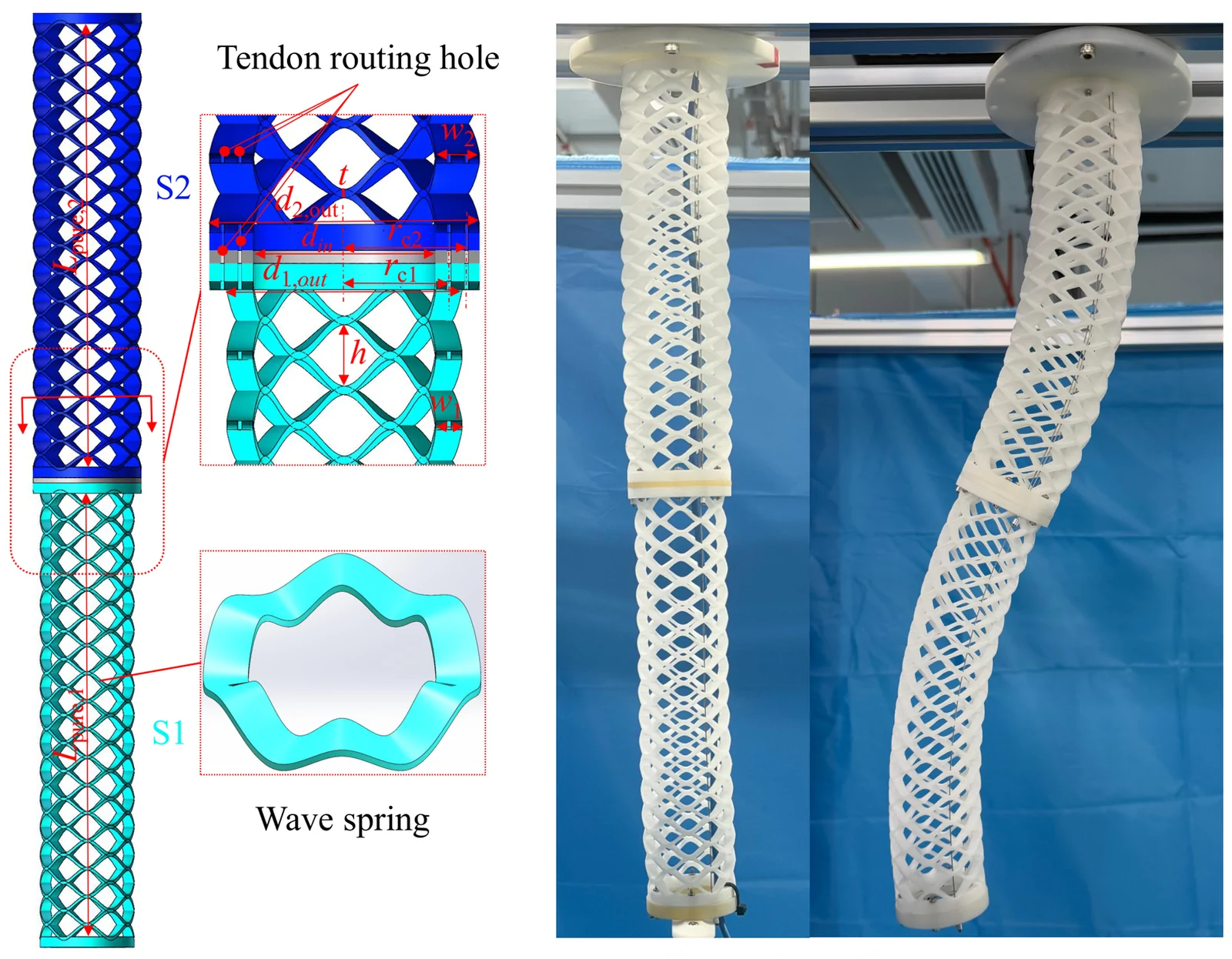
Structural design and assembled prototype of the bioinspired soft manipulator based on wave spring architectures.

Taking the elephant trunk as the bionic prototype, this soft manipulator is designed as a two-stage segmented structure to replicate the flexible bending capability of biological elephant trunks. The overall system consists of two serial modular wave spring segments (denoted S1 and S2), connecting flanges, upper/lower end caps, and distributed tendon routing holes for tendon-driven actuation, as visualized in Figure 1. The design of the two-segment wave-spring manipulator draws biological inspiration from the biological characteristics of elephant trunks. An elephant trunk exhibits typical gradient-stiffness mechanical properties: its proximal segments possess higher stiffness to undertake load-bearing tasks, whereas the distal segments are more flexible to accomplish delicate manipulation tasks. Inspired by such gradient-stiffness characteristics, our manipulator adopts different rib widths for the lower segment S1 and upper segment S2, generating a gradient-stiffness distribution along the robot body. Moreover, the serial modular segmented layout of our prototype mimics the hierarchical muscular arrangement of natural elephant trunks.

Figure 1 integrates the parametric CAD schematic and the corresponding 3D-printed physical prototype. The left CAD view marks all core geometric variables of the two-stage wave spring skeleton: $H_{i}$ represents the total axial length of each segment; *h* denotes the vertical wave peak-to-trough height of a single wave unit; *t* is the uniform wall thickness of the wave spring profile; and $w_{1}$ and $w_{2}$ separately define the lateral rib widths of S1 and S2 to realize stiffness differentiation. The inner and outer diameters of each wave spring skeleton are labeled as $d_{i,\mathrm{in}}$ and $d_{i,\mathrm{out}}$, while the tendon routing holes are evenly distributed on the intermediate connecting flange, with distinct hole radii $r_{\mathrm{hole},1}$ and $r_{\mathrm{hole},2}$ set for S1 and S2. The right side of Figure 1 displays the fabricated prototype, where both the upper segment S2 and lower segment S1 are integrally manufactured via fused deposition modeling (FDM) with flexible TPU material. The printed manipulator is presented under two typical configurations, the straight neutral state and passive omnidirectional bending deformation, which intuitively verifies the compliant motion capability of the wave spring architecture. The integral one-step 3D printing process eliminates assembly gaps between wave units, guarantees consistent wall thickness t=2mm across the entire skeleton, and maintains the preset gradient-stiffness characteristic differentiated by rib widths $w_{1}$ and $w_{2}$ for S1 and S2. Flexible TPU filament is selected as the printing material to balance sufficient elastic recoverability and structural rigidity, so the wave spring skeleton can rebound to its original straight shape after releasing external bending loads without permanent plastic deformation.

All the precise geometric dimensions of the two segments are summarized in Table 1, with all units in millimeters. S1 (the lower segment) adopts narrower wave ribs ($w_{1}=6\;\mathrm{mm}$) and a smaller outer diameter ($d_{1,\mathrm{out}}=53.72\;\mathrm{mm}$), equipped with tendon routing holes of radius $r_{\mathrm{hole},1}=24\;\mathrm{mm}$. S2 (the upper segment) uses wider wave ribs ($w_{2}=10\;\mathrm{mm}$) and a larger outer diameter ($d_{2,\mathrm{out}}=61.72\;\mathrm{mm}$), with a tendon hole radius of $r_{\mathrm{hole},2}=28\;\mathrm{mm}$. Such dimensional differences produce gradient stiffness along the robot’s length, matching the variable flexibility characteristic of elephant trunks. Each segment has a total length of $H_{1}=H_{2}=266\;\mathrm{mm}$ (including $a_{iu}=a_{id}=6\;\mathrm{mm}$ planar end caps at both ends), and the pure deformable wave spring length reaches $L_{\mathrm{pure},i}=254\;\mathrm{mm}$, containing 16 pairs of periodic wave units. The vertical gap between wave crests and troughs is fixed at h=14mm, and the wall thickness of all wave sheets is uniformly t=2mm. The straight connecting flange between S1 and S2 has an axial length of $L_{\mathrm{flange}}=15\;\mathrm{mm}$, which serves as the assembly carrier for tendon routing holes and realizes serial connection of the two segments.

As for the axial compression ratio of the wave-spring soft manipulator, the periodic wave-shaped topology of the wave spring skeleton reserves discrete axial compressible gaps between adjacent wave units, which determine the maximum axial contraction capacity of the entire soft manipulator. Under the ideal geometric condition without material deformation and assembly clearance, the total maximum compressible stroke of the whole manipulator is the sum of the vertical wave gaps of all periodic wave units, expressed as

(1) $$\Delta L_{\max}=h\cdot N_{\mathrm{wave}}$$

where *h* is the vertical peak-to-trough height of a single wave unit, and $N_{\mathrm{wave}}$ denotes the total number of wave pairs of a single segment.

Define the total original full length of the two-segment manipulator as $L_{\mathrm{total},\mathrm{ori}}$, which sums the pure deformable length, upper/lower end cap height of each segment and the intermediate connecting flange length:

(2) $$L_{\mathrm{total},\mathrm{ori}}=L_{\mathrm{pure},1}+a_{1u}+a_{1d}+L_{\mathrm{pure},2}+a_{2u}+a_{2d}+L_{\mathrm{flange}}$$

After full axial compression, the minimum overall length of the manipulator is $L_{\mathrm{total},\min}=L_{\mathrm{total},\mathrm{ori}}-\Delta L_{\max}$. The axial compression ratio $R_{L}$ is defined as the ratio of the original full length to the compressed minimum length, which quantitatively characterizes the telescopic performance of the soft manipulator:

(3) $$R_{L}=\frac{L_{\mathrm{total},\mathrm{ori}}}{L_{\mathrm{total},\mathrm{ori}}-h\cdot N_{\mathrm{wave}}}$$

Substituting the structural geometric parameters listed in Table 1 for quantitative calculation: $R_{L}\approx 1.693$.

**Figure 2:**
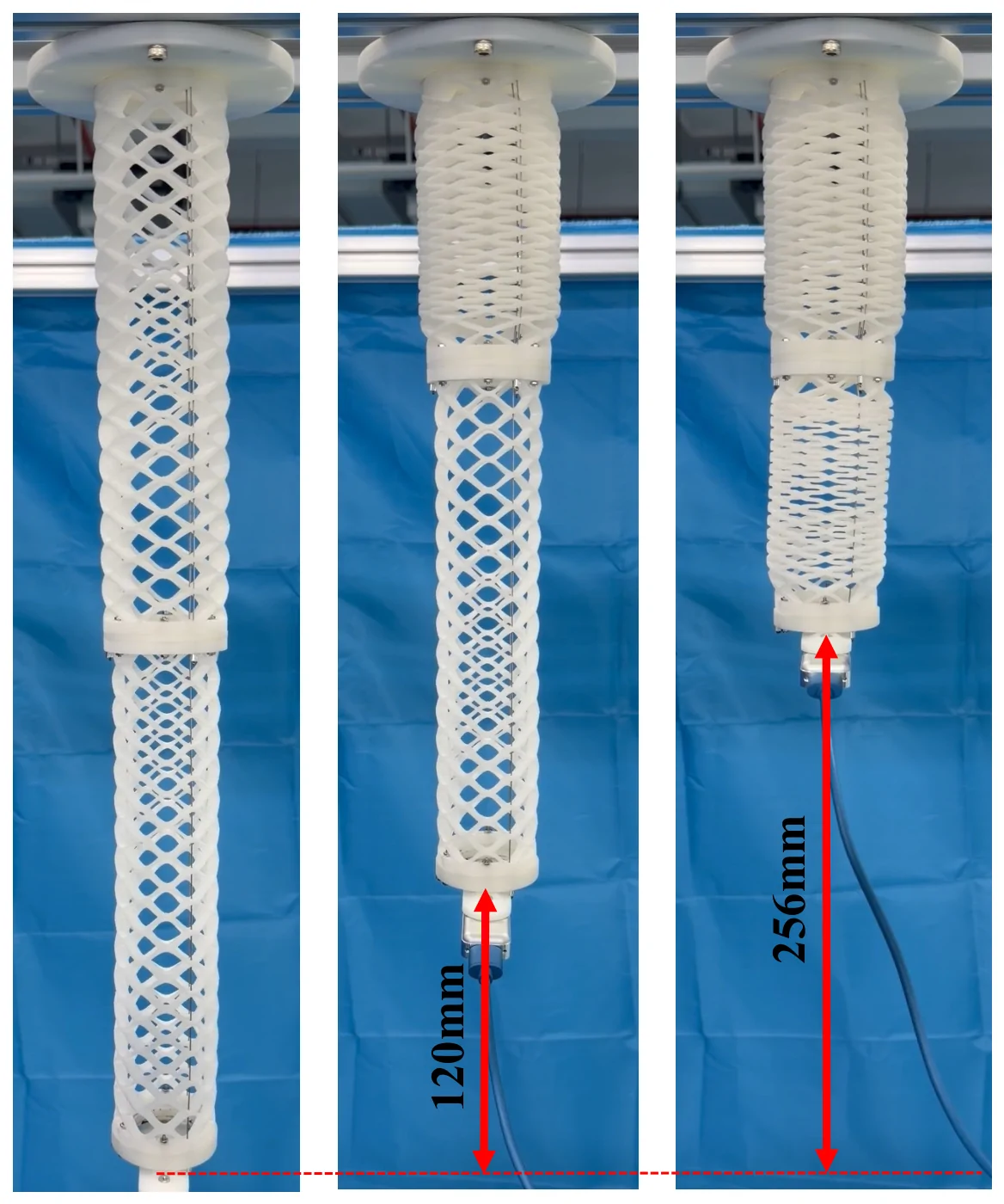
Axial compression test of the two-segment wave-spring soft manipulator. From left to right: original unloaded neutral state; intermediate compression state, with a 120 mm contraction stroke of upper segment S2; ultimate compression state, where S2 is compressed by 120 mm and lower segment S1 is compressed by 136 mm, achieving a total axial contraction stroke of 256 mm.

The theoretical compression ratio $R_{L}\approx 1.693$ indicates that the overall length of the two-segment wave spring manipulator can be reduced to 1/1.693≈59.1% of the original full length under ideal geometric compression, demonstrating excellent axial telescopic capacity. It should be noted that this theoretical value ignores the viscoelastic creep of TPU material, friction between driving tendons, and structural contact interference; the actual compression ratio measured by the physical prototype will deviate slightly from this ideal theoretical result. An axial compression experiment with the fabricated integral TPU two-segment wave spring manipulator was carried out to verify its telescopic performance, as shown in Figure 2. Three driving tendons are uniformly arranged circumferentially across each segment and penetrate through the reserved tendon routing holes for independent actuation; the feed length of each tendon is precisely regulated by a separate servo motor.

## 3. Control of the Wave-Spring Soft Manipulator

**Figure 3:**
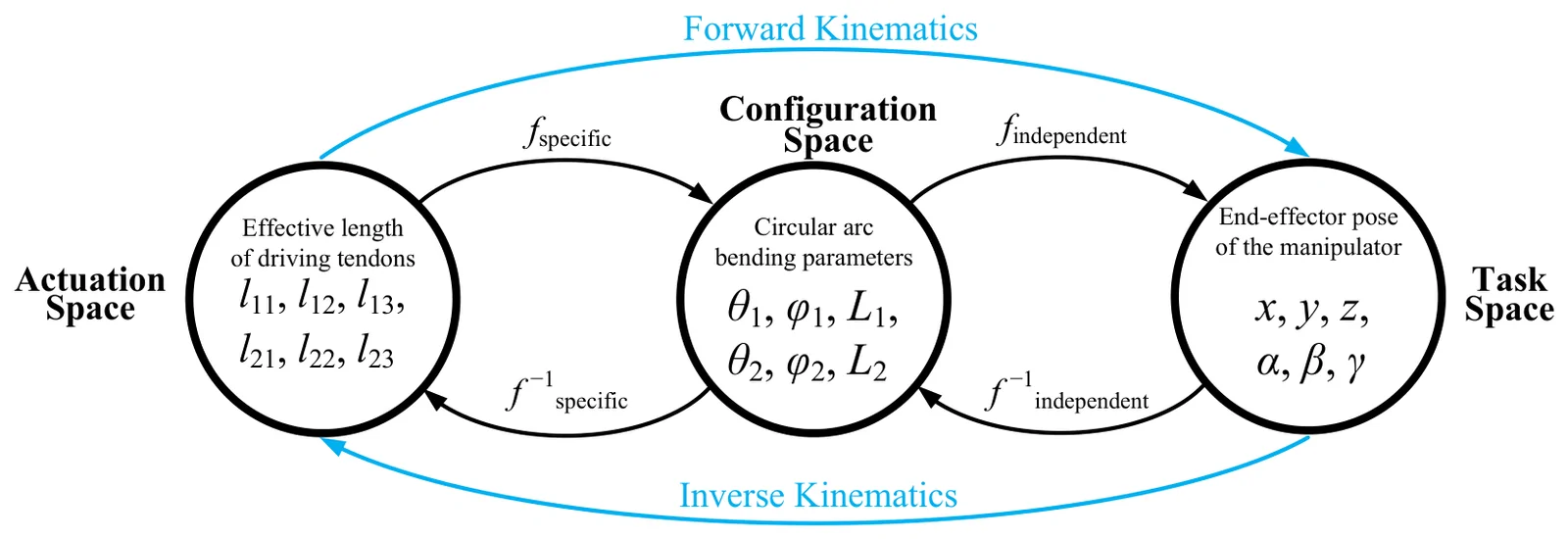
Three-space kinematic mapping diagram of continuum soft manipulator, illustrating the forward and inverse transformation loops covering actuation space, configuration space, and task space.

The mapping relationships among the three core spaces of the two-segment wave-spring soft manipulator are visualized in Figure 3. For traditional continuum robot kinematic modeling, the configuration space is introduced as an intermediate bridge to connect the actuation space and the task space, which enables the derivation of forward kinematics and inverse kinematics transformation models step by step.

In the actuation space, the state variables are the effective feed lengths of six circumferentially distributed driving tendons: $l_{11},l_{12},l_{13},l_{21},l_{22},l_{23}$. The intermediate configuration space adopts circular-arc constant-curvature parameters $\theta_{i},\varphi_{i},L_{i}$ (i=1,2) to describe the bending shape of each serial segment. The task space characterizes the 6-DoF end pose with Cartesian position x,y,z and Euler rotation angles α,β,γ. The forward mapping $f_{\mathrm{specific}}$ and $f_{\mathrm{independent}}$ constitute the overall forward kinematics from tendon lengths to terminal pose, while the inverse functions $f_{\mathrm{specific}}^{-1}$ and $f_{\mathrm{independent}}^{-1}$ realize the reverse solution, from target pose to required tendon lengths.

However, the model-based kinematic framework built on the constant-curvature assumption has several unavoidable defects. First, the circular-arc geometric approximation deviates from the actual deformation of the soft manipulator, which reduces the pose prediction accuracy. Second, the analytical model cannot fully compensate external disturbances, tendon friction and material viscoelastic creep, leading to weak anti-interference performance and poor robustness. Third, the multi-layer geometric transformation brings heavy computational overhead for real-time control. Most importantly, there exists no closed-form analytical inverse kinematic solution for a two-or-more-stage serial soft manipulator.

To address the above bottlenecks, learning-based model-free kinematic strategies have been widely investigated in recent research. This work adopts a model-free data-driven scheme, where a neural network is trained to directly approximate the inverse kinematics mapping of the wave-spring soft manipulator without relying on geometric analytical derivation.

### 3.1. Neural Network-Based Inverse Kinematics Model

#### 3.1.1. Training Data Acquisition

Neural network algorithms are categorized as data-driven modeling approaches which rely on experimental datasets to establish the mapping between the actuation space and task space of the soft manipulator. Therefore, the first step is to collect massive sample data covering the full workspace of the manipulator via exhaustive motion traversal, where the direct control input during data sampling refers to motor encoder positions.

The detailed data collection procedure is described as follows. The total encoder variation corresponding to the manipulator’s stroke from its fully compressed shortest state to maximum extension is evenly divided into four equal intervals Δl, yielding five discrete encoder positions: 0,Δl,2Δl,3Δl,4Δl. During traversal, only one single motor is actuated to shift its encoder value by Δl at each step. Once the arm’s deformation stabilizes, the relative encoder increments of all six driving motors with respect to the initial position are recorded. Meanwhile, an Intel RealSense T265 tracking camera mounted at the distal end of the manipulator synchronously captures the 6-DoF end pose of the soft manipulator. The T265 camera outputs pose data at a sampling rate of 200 Hz, with a typical drift of less than 1% of the traveled distance in indoor environments with abundant visual features.

**Figure 4:**
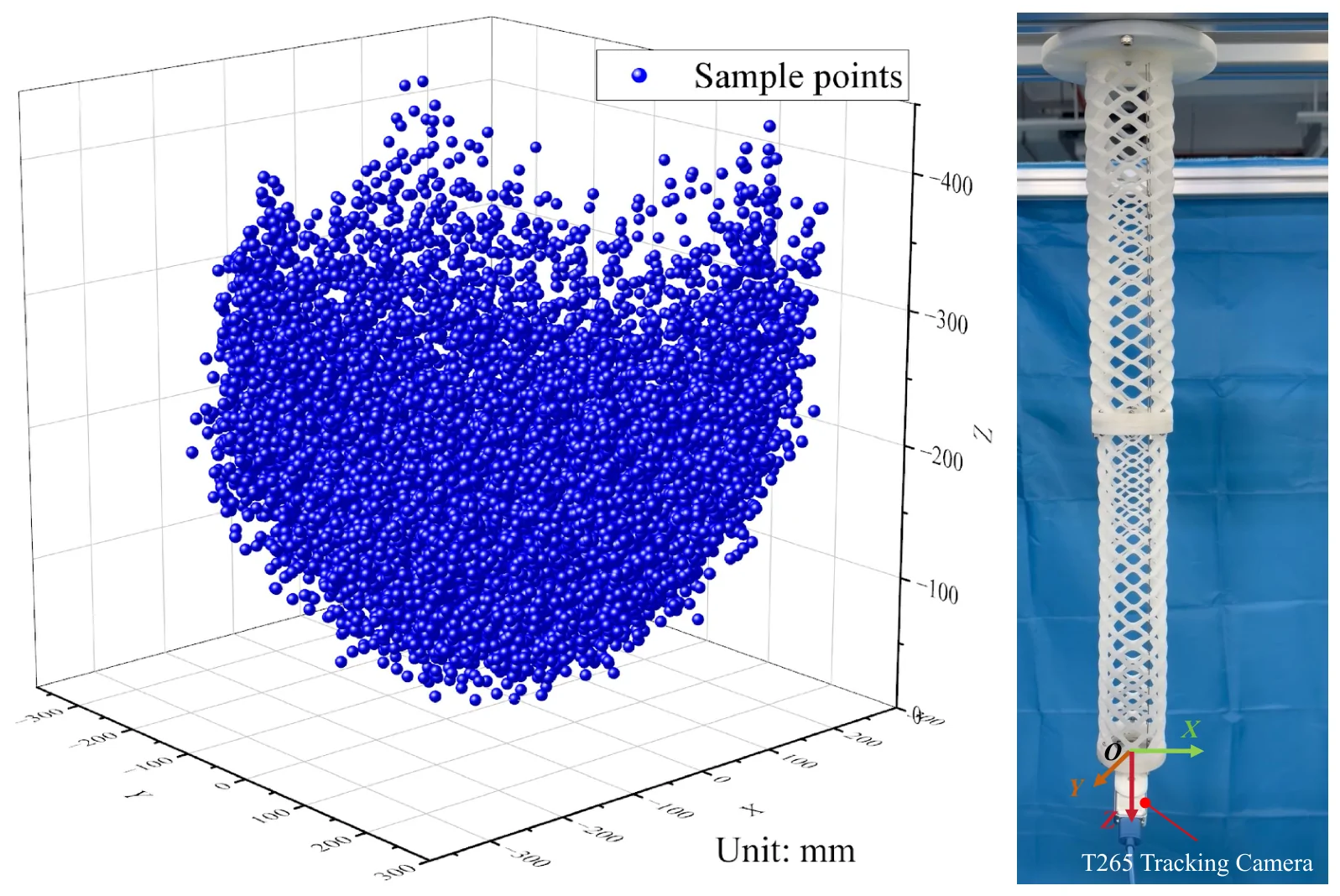
Spatial distribution of valid training data points of the two-segment wave-spring soft manipulator.

Based on this full-factorial traversal strategy, the theoretical total number of sample points is $5^{6}$ = 15,625. However, raw data contains some invalid outliers caused by structural jitter, camera tracking loss, and assembly clearance, which are manually filtered according to the spatial continuity rule: adjacent sampling points should exhibit continuous and regular pose variation within the manipulator’s workspace. After outlier elimination, 15,078 valid data samples are retained, and the spatial distribution of these samples is visualized in Figure 4. To intuitively characterize the terminal displacement of the soft manipulator, the native coordinate frame OXYZ of the T265 tracking camera mounted on the manipulator’s end under the initial straight configuration is defined as the unified reference coordinate system for all motion experiments, as illustrated in Figure 4.

We define explicit criteria for removing invalid training samples. Considering the physical characteristics of the soft manipulator, consecutive sampling points are supposed to maintain continuous and regular pose variation within the manipulator’s workspace. Samples suffering from abrupt and physically unreasonable pose jumps, which are mainly induced by camera tracking loss, structural jitter, and assembly clearance, are regarded as outliers and discarded from the training dataset.

#### 3.1.2. Construction of Neural Network Model

To construct the data-driven inverse kinematics model for the two-segment wave-spring continuum manipulator, the input and output dimensions of the neural network must be defined first; these correspond to the input layer and output layer of the neural network, respectively.

During the deformation of the designed soft manipulator, the rotation angle γ around the Z-axis is uncontrollable and strongly coupled with x,y,z,α,β. Therefore, the Z-axis rotation γ is discarded in the inverse kinematics mapping. The input of the network is defined as the 5-dimensional end pose vector (x,y,z,α,β), while the network output corresponds to the effective elongation of the six driving tendons $\Delta l_{11}$, $\Delta l_{12}$, $\Delta l_{13}$, $\Delta l_{21}$, $\Delta l_{22}$, and $\Delta l_{23}$, represented by the relative encoder increments of the six driving motors. Accordingly, the input layer contains 5 neurons, and the output layer contains 6 neurons. All samples collected via exhaustive traversal are preprocessed by removing the Z-axis rotation term γ to form the training dataset of the neural network.

The neural network model is built based on TensorFlow framework under the Python 3.13.5 environment. The full dataset is randomly split into a training set and a test set at a ratio of 8.5:1.5. To eliminate the adverse impact of abnormal sample data, the input features of the training set are standardized via Equation (4) to follow a standard normal distribution with zero mean and unit variance:

(4) $$t^{\prime }=\frac{t-\mu }{\sigma }$$

where $t^{\prime }$ denotes the normalized input features of the training set, i.e., $x_{1},x_{2},x_{3},x_{4},x_{5}$; *t* represents the raw sampled pose data x,y,z,α,β; μ is the mean value of each input dimension, namely, $\mu_{x},\mu_{y},\mu_{z},\mu_{\alpha },\mu_{\beta }$; and σ is the standard deviation of each input dimension, namely, $\sigma_{x},\sigma_{y},\sigma_{z},\sigma_{\alpha },\sigma_{\beta }$.

After repeated comparative experiments, the network architecture adopted in this work is specified as follows: one input layer with 5 neurons, three fully connected hidden layers with 256, 128 and 64 neurons, respectively, and one fully connected output layer with 6 neurons. The GELU activation function is adopted for all hidden layers, and linear activation is applied to the output layer to support unrestricted continuous tendon-length prediction. L2 regularization and dropout layers with a dropout rate of 0.25 are embedded after each hidden layer to suppress overfitting, and batch normalization is implemented to accelerate convergence.

The Adam optimizer is selected with an initial learning rate of 0.0003. Instead of the traditional mean squared error (MSE), the Huber loss function with δ=1.0 is utilized to enhance robustness against outliers in the dataset. Three training callbacks are configured to optimize training efficiency: 1. Early stopping: Training terminates if the validation loss fails to decrease within 15 consecutive epochs, and the optimal weights are restored automatically; 2. Learning rate reduction: The learning rate decays by half when the validation loss plateaus for 8 epochs, with a lower bound of $1\times 10^{-6}$; 3. Model checkpoint: The network weights with the minimum validation loss are automatically saved during training.

**Figure 5:**
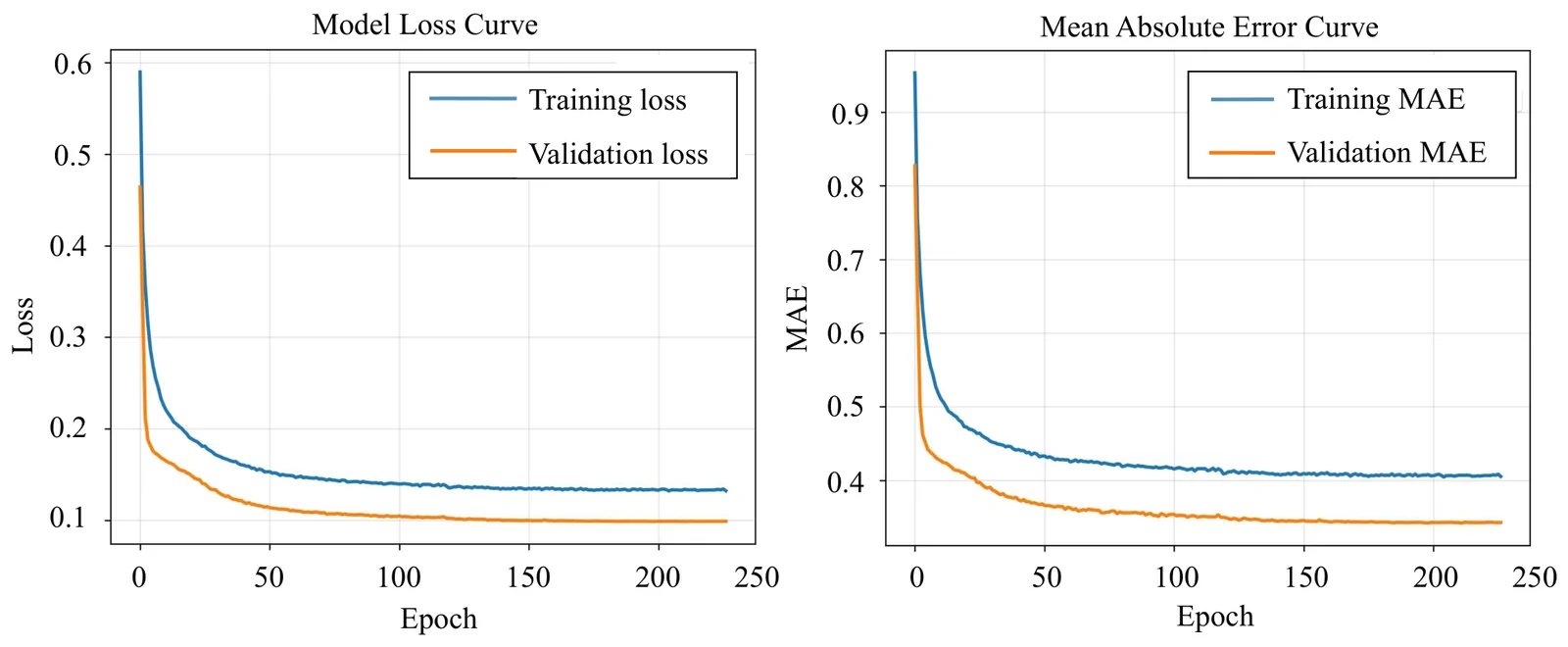
Variation curves of training loss and validation loss of the inverse kinematics neural network.

The training epoch upper limit is set to 500 with a batch size of 64. The structural hyperparameters and training performance of the proposed neural network are summarized in Table 2. The evolution curves of training loss and validation loss during the training process are illustrated in Figure 5.

After training, the standardization transformers for the input pose and output tendon lengths are saved for offline inference. A dedicated prediction function is developed to receive the 5-D target pose (x,y,z,α,β) and output the corresponding six tendon-length variations directly, which provides feedforward reference signals for the subsequent iterative learning control module.

#### 3.1.3. Performance Test of the Inverse Kinematics Model

To verify the accuracy of the trained inverse kinematics model based on a neural network, a trajectory-tracking experiment was carried out. The target trajectory was a circle with a radius of 100mm lying on the plane of z=−100mm, whose center was located on the *Z*-axis. The attitude of all target points was fixed at α=β=0. A total of 100 equally spaced points sampled on the circle were set as the desired end poses of the soft manipulator.

**Figure 6:**
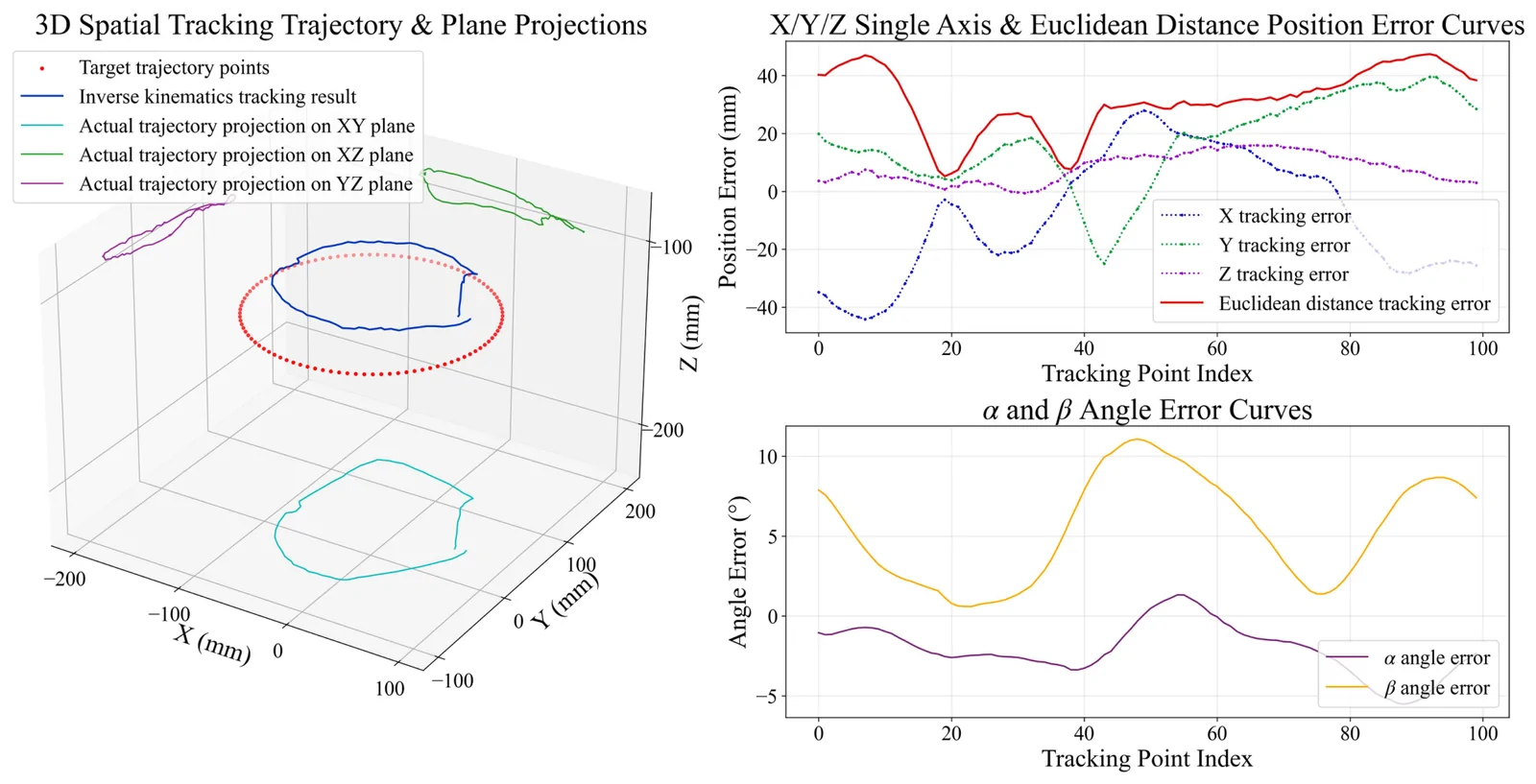
The 3D spatial trajectory, planar projection and dimensional tracking errors for circular tracking based on pure neural network inverse kinematics.

In the experiment, the pre-generated target point sequence, trained neural network inverse kinematics model, and normalization parameters were loaded first. Each 5-dimensional target pose was normalized using the pre-fitted scaler and fed into the neural network to predict the incremental lengths of the six driving tendons, which were then transmitted to the servo motors for motion control. The overall tracking performance is illustrated in Figure 6. The projection of the actual tracking trajectory on the XY plane roughly matches the profile of the target circle, yet persistent tracking errors exist along the *X* and *Y* axes. Moreover, the projections on the XZ and YZ planes reveal prominent *Z*-direction deviations. In general, the pure data-driven inverse kinematics model cannot achieve satisfactory tracking accuracy for circular trajectories.

The tracking errors of each dimension are plotted in Figure 6. The Euclidean distance error between the actual and target end positions remains above 6mm throughout the whole tracking process, with a maximum value close to 50mm. Meanwhile, non-negligible angular errors about the *X* and *Y* axes are observed, whose peak value exceeds $10^{\circ }$. The experimental results demonstrate that the proposed neural network-based inverse kinematics model suffers from low precision in both position and attitude tracking.

Nevertheless, it can be observed from the spatial trajectory and its XY projection that the relative spatial relationship between consecutive tracked points is consistent with that of the corresponding target points. In other words, the overall movement trend of the manipulator matches the target trajectory well, whereas static point-to-point offset errors are inevitable. It can be concluded that the neural network can roughly capture the intrinsic inverse kinematic mapping of the manipulator. However, the poor single-point prediction accuracy limits its control performance.

Even with a large-scale training dataset, the open-loop prediction accuracy of the neural network-based inverse kinematics is restricted by multiple non-ideal factors. First, the viscoelastic hysteresis and time-dependent creep effect of the TPU material produce pose drift even under identical tendon-length inputs, introducing intrinsic input--output uncertainty into the collected dataset. Second, assembly clearances at tendon routing holes and friction between tendons and connecting flanges bring unmodeled external disturbances. Third, sparse sampling near the workspace boundaries results in deteriorated fitting performance for boundary poses. These practical factors lead to inevitable open-loop prediction errors, which further indicates that additional error compensation strategies are indispensable for high-precision trajectory tracking.

### 3.2. Single-Point Positioning Based on Iterative Learning Control

Although the inverse kinematics model obtained via a neural network can roughly reflect the motion characteristics of the tendon-driven soft manipulator, substantial tracking deviations exist between the actual end position and the desired target, which prevents high-precision positioning. A T265 tracking camera mounted at the manipulator end can provide real-time feedback of the end pose, which can be utilized to construct closed-loop feedback control and improve motion accuracy.

The control input of the manipulator system consists of six tendon-length variations $\Delta l_{11}$, $\Delta l_{12}$, $\Delta l_{13}$, $\Delta l_{21}$, $\Delta l_{22}$, and $\Delta l_{23}$, while the system output is a 5-dimensional end pose (x,y,z,α,β). Conventional PID control cannot be directly applied due to the strong nonlinearity of the soft manipulator. On this basis, this paper proposes a closed-loop motion control framework integrated with iterative learning control on top of the data-driven inverse kinematics model to achieve high-precision positioning of the two-segment continuum manipulator.

#### 3.2.1. Principle of Iterative Learning Control

Iterative learning control improves tracking performance by repeatedly executing control tasks and updating control signals using the error between measured outputs and reference trajectories. Under ideal conditions, the steady-state tracking error converges to zero as the number of iterations increases. A comprehensive theoretical introduction of iterative learning control can be found in [30]; this section only elaborates the iterative learning control updating law adopted in this work.

For the proposed continuum manipulator system, define the 6-dimensional control vector of tendon-length variations as u(n)=$(\Delta l_{11}$, $\Delta l_{12}$, $\Delta l_{13}$, $\Delta l_{21}$, $\Delta l_{22}$, $\Delta l_{23})$ and the 5-dimensional measurable end pose output vector as y(n)=(x,y,z,α,β), where *n* = 1, 2, 3, … denotes the index of discrete target points. The data-driven inverse kinematics mapping is expressed as

(5) $$u\left(n\right)=f_{\mathrm{IK}}\left(y(n)\right)$$

where $f_{\mathrm{IK}}\left(\cdot \right)$ represents the neural network-based inverse kinematics model, which remains invariant during repetitive manipulator motions and satisfies the repetitive property required by iterative learning control.

Let k=0,1,2,… denote the iteration number for single-point repetitive positioning. $u_{k}\left(n\right)$ and $y_{k}\left(n\right)$ represent the control input and measured end pose at the *k*-th iteration for the *n*-th target point, respectively. The repetitive inverse kinematics mapping can be rewritten as

(6) $$u_{k}\left(n\right)=f_{\mathrm{IK}}\left(y_{k}\left(n\right)\right)$$

The output error vector between the desired and measured pose is defined as

(7) $$e_{k}\left(n\right)=y_{d}\left(n\right)-y_{k}\left(n\right)$$

where $y_{d}\left(n\right)$ stands for the reference desired pose of the *n*-th target point.

According to Equation (5), the control input u is uniquely determined by the target pose $y_{d}$. Therefore, the P-type learning law is transformed into a reference-update form with modified desired pose $y_{d}^{k}\left(n\right)$:

(8) $$y_{d}^{k+1}\left(n\right)=y_{d}^{k}\left(n\right)+Le_{k}\left(n\right)$$

where the tracking error is redefined as $e_{k}\left(n\right)=y_{d}^{0}\left(n\right)-y_{k}\left(n\right)$, where $y_{d}^{0}\left(n\right)$ is the fixed ideal reference pose of the *n*-th target throughout all iterations.

Two termination criteria are set to stop iterative updates after each iteration:

(1) Tolerance error threshold constraint:
  (9) $$∥y_{d}^{0}\left(n\right)-y_{k}\left(n\right)∥<\varepsilon$$
(2) Maximum iteration limit constraint:
  (10) k>ν

where ε is the allowable tracking error bound, and ν denotes the preset maximum number of iterations.

**Figure 7:**
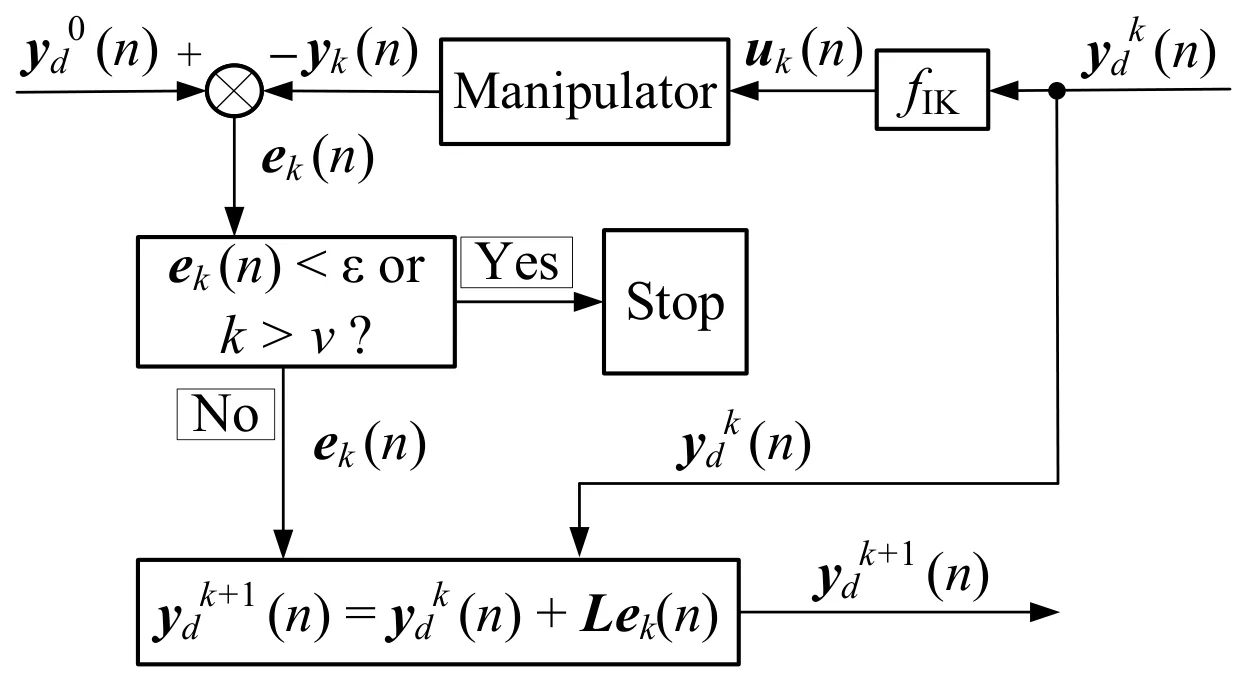
Flowchart of the proposed iterative learning control algorithm.

Combining Equations (5)–(10), the complete flow chart of the proposed iterative learning control algorithm is illustrated in Figure 7. In the flowchart, $y_{d}^{k}\left(n\right)$ represents the modified desired pose at the *k*-th iteration, and $y_{k}\left(n\right)$ is the actual end pose captured by the T265 tracking camera after the *k*-th movement. The original ideal reference pose $y_{d}^{0}\left(n\right)$ remains unchanged during the whole iterative process, and the initial control signal is calculated as $u_{0}\left(n\right)=f_{\mathrm{IK}}\left(y_{d}^{0}\left(n\right)\right)$.

#### 3.2.2. Single-Point Positioning Experiment

**Figure 8:**
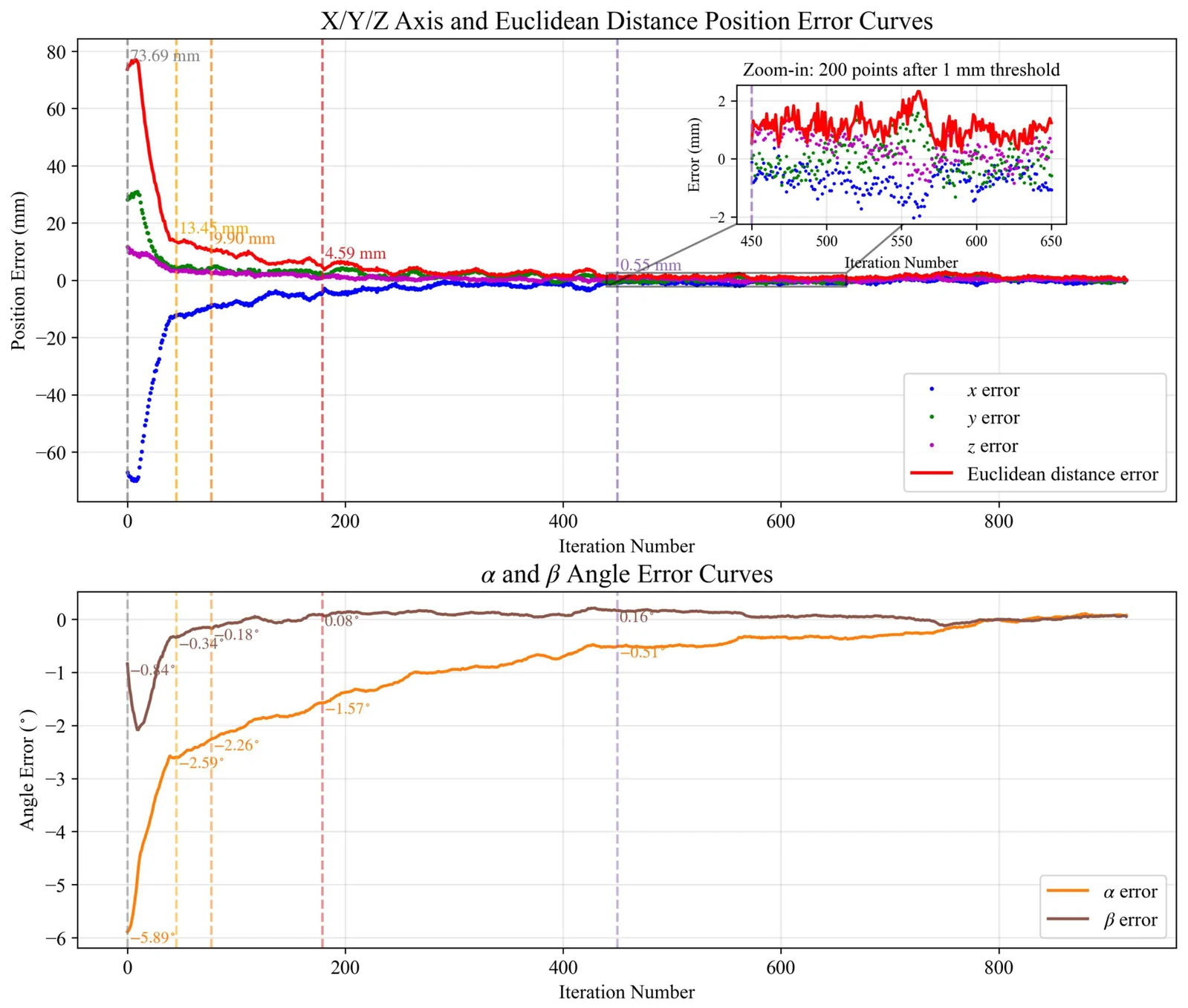
Tracking error curves of single-point positioning within the first 900 iterations.

To verify the effectiveness of the proposed control method, single-point positioning experiments are carried out on the soft manipulator. The desired target pose is selected as (x=100,y=100,z=−100,α=0,β=0). At the initial state of the continuum manipulator, the desired end output $y_{d}^{0}=\left(100,\;100,\;-100,\;0,\;0\right)$ is assigned. Subsequently, the control system feeds $y_{d}^{0}$ into the neural network-based inverse kinematics model to generate the initial motor control command $u_{0}=f_{\mathrm{IK}}\left(y_{d}^{0}\right)$. After the motors stabilize at the position corresponding to $u_{0}$, the iterative learning control scheme illustrated in Figure 7 is adopted to regulate the motion of the soft manipulator, with one iteration executed per control cycle. The error data collected from the first 900 iterative control steps are plotted in Figure 8.

Figure 8 illustrates the tracking-error evolution of the single-point positioning experiment. The initial Euclidean distance error produced by the neural network-based inverse kinematics model reaches 73.695mm. The error drops rapidly to 13.454mm after 45 iterations. The Euclidean distance error decreases to 9.896mm at the 77th iteration, and further reduces to 4.589mm at the 179th iteration. It takes 450 iterations for the Euclidean error to converge below 1mm, where the corresponding angular errors are $0.157^{\circ }$ for β and $-0.509^{\circ }$ for α. The zoom-in subplot visualizes the error performance over the subsequent 200 iterations after the error falls below 1mm. It can be observed that the Euclidean distance error fluctuates mainly within the range of ±2mm during this period. In general, the tracking error remains within this narrow band for the following 300 iterations. These results demonstrate that the combination of the neural network-based inverse kinematics and iterative learning control achieves high-precision steady-state positioning performance for the soft manipulator.

#### 3.2.3. Anti-Disturbance Performance Test

In practical task execution, continuum manipulators are inevitably subjected to various uncertain disturbances induced by external environments. In particular, contact disturbances between obstacles and the manipulator will lead to deviations of the end pose from the desired pose. Therefore, disturbance experiments are carried out to investigate whether the iterative learning control strategy can re-achieve precise end tracking after external perturbation, so as to verify the anti-disturbance robustness of the proposed control algorithm.

**Figure 9:**
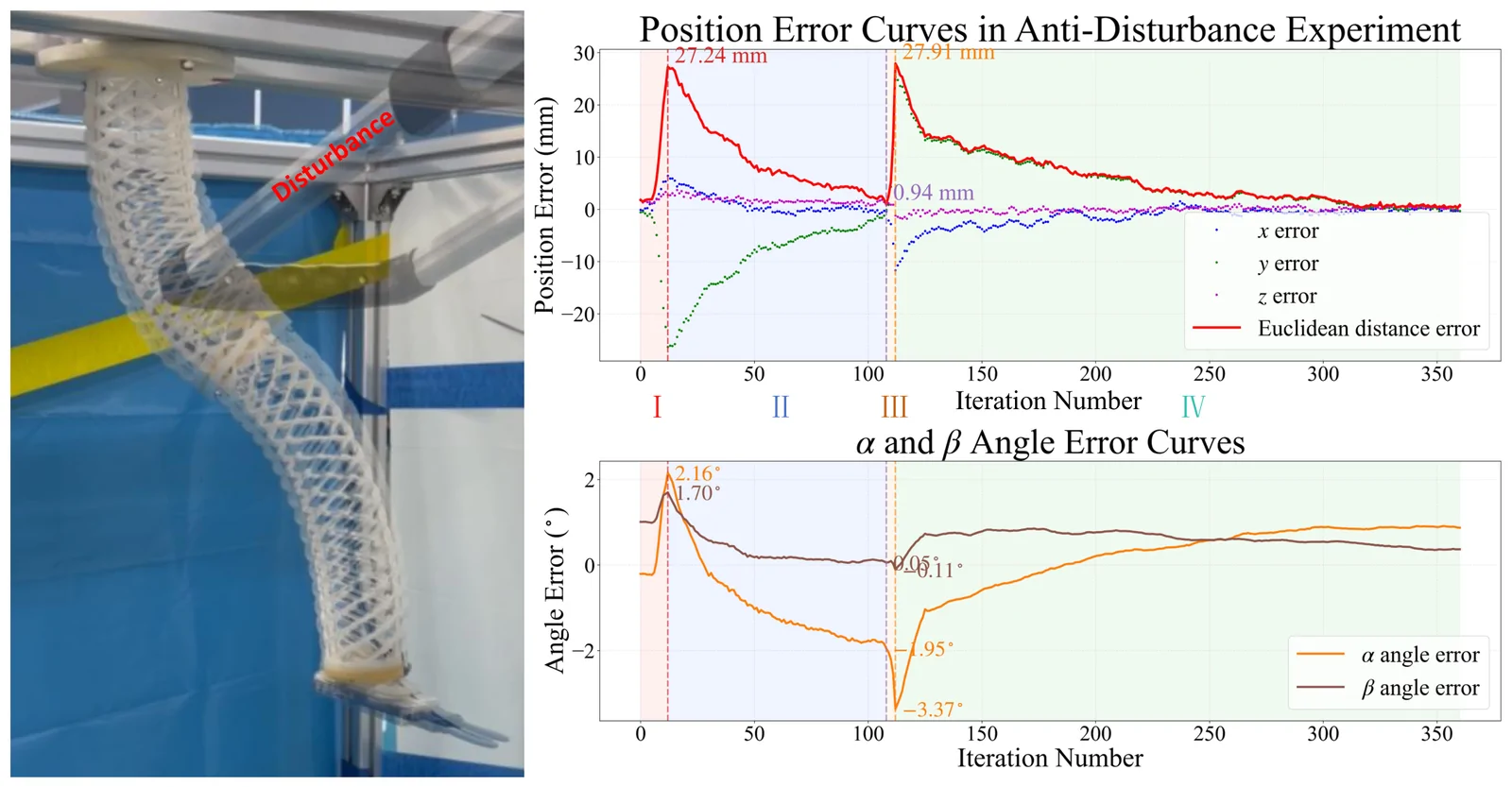
Disturbance experiment of the soft manipulator. **Left**: Configuration comparison between undisturbed state and disturbed repositioning state after contact perturbation; **Right**: Four-stage tracking error curves under disturbance loading and disturbance removal.

The target pose is set as (x=100,y=100,z=−100,α=0,β=0), and the manipulator is maintained at the tracking state via iterative learning control until the positioning error stabilizes within 2mm. Afterwards, a metal rod is adopted as the external obstacle to push and fix the soft manipulator body to introduce sustained mechanical disturbance. As illustrated in Figure 9, the left subfigure compares the overall configurations of the undisturbed manipulator and the disturbed manipulator after being pushed (Phase I). The right subfigure plots the evolution of position tracking errors throughout the entire process: the error variation during iterative correction under sustained disturbance (Phase II), and the error rebound (Phase III) and re-convergence (Phase IV) after the obstacle is removed (obstacle removal introduces a new transient disturbance by breaking the original balanced configuration).

The experimental results demonstrate that the manipulator can accurately track the target pose via iterative learning control with adjusted actuation parameters even when its whole configuration is deformed by external disturbance. Such performance is difficult to realize by pure kinematic model-based control schemes. It further verifies the effectiveness and strong anti-disturbance robustness of the iterative learning control method for continuum manipulators.

### 3.3. Trajectory Tracking Based on Iterative Learning Control

#### 3.3.1. Iterative Learning Control Scheme for Trajectory Tracking

The experimental results shown in Figure 8 demonstrate that the iterative learning control learning law given by Equation (8) can achieve high-precision tracking of a single-point target. When a prescribed target trajectory needs to be tracked, the target trajectory can be discretized into *N* adjacent and ordered target points according to the periodic control characteristics of the continuum manipulator. The tracking of the entire target trajectory can then be achieved by sequentially tracking these *N* ordered target points.

However, the initial control vector used in the iterative learning control method for single-point target tracking is given by

(11) $$u_{0}\left(n\right)=f_{\mathrm{IK}}\;\left(y_{d}^{0}\left(n\right)\right),$$

which is obtained directly from the inverse kinematics model based on the neural network. As shown in Figure 6, the inverse kinematics error is relatively large. Therefore, if the tracking of each target point is regarded as an independent repetition of the single-point tracking process shown in Figure 8, the error between the actual position and the target point can eventually be reduced to a small value after several iterations. Nevertheless, the tracking process for every target point would start with a large initial error and then gradually converge, as illustrated in Figure 6. Consequently, this strategy cannot provide the desired high-precision tracking performance for the entire target trajectory.

To ensure that the error between the end of the soft manipulator and the tracking trajectory remains as small as possible throughout the trajectory-tracking process, the iterative control learning law in Equation (8) is appropriately modified to achieve continuous, high-precision tracking of adjacent points on the target trajectory. Consider two consecutive target points *n* and n+1. Once the tracking of target point *n* satisfies the iteration termination condition, the desired target pose $y_{d}^{0}\left(n+1\right)$ corresponding to target point n+1 can be directly used to replace the desired target pose $y_{d}^{0}\left(n\right)$ corresponding to target point *n* in the iterative learning control law in Equation (8), where

(12) $$e_{k}\left(n\right)=y_{d}^{0}\left(n\right)-y_{k}\left(n\right).$$

The iterative control learning law for trajectory tracking can therefore be obtained, in conjunction with Figure 7, as

(13) $$\left\{\begin{array}{cccc} y_{d}^{k+1}\left(n\right)\; & =y_{d}^{k}\left(n\right)+L\left(y_{d}^{0}\left(n\right)-y_{k}\left(n\right)\right),\; & \; & ∥y_{d}^{0}\left(n\right)-y_{k}\left(n\right)∥>\varepsilon \;\mathrm{or}\;k<\nu ,\\ y_{d}^{1}\left(n+1\right)\; & =y_{d}^{k}\left(n\right)+L\left(y_{d}^{0}\left(n+1\right)-y_{k}\left(n\right)\right),\; & \; & ∥y_{d}^{0}\left(n\right)-y_{k}\left(n\right)∥\leq \varepsilon \;\mathrm{or}\;k\geq \nu . \end{array}\right.$$

**Figure 10:**
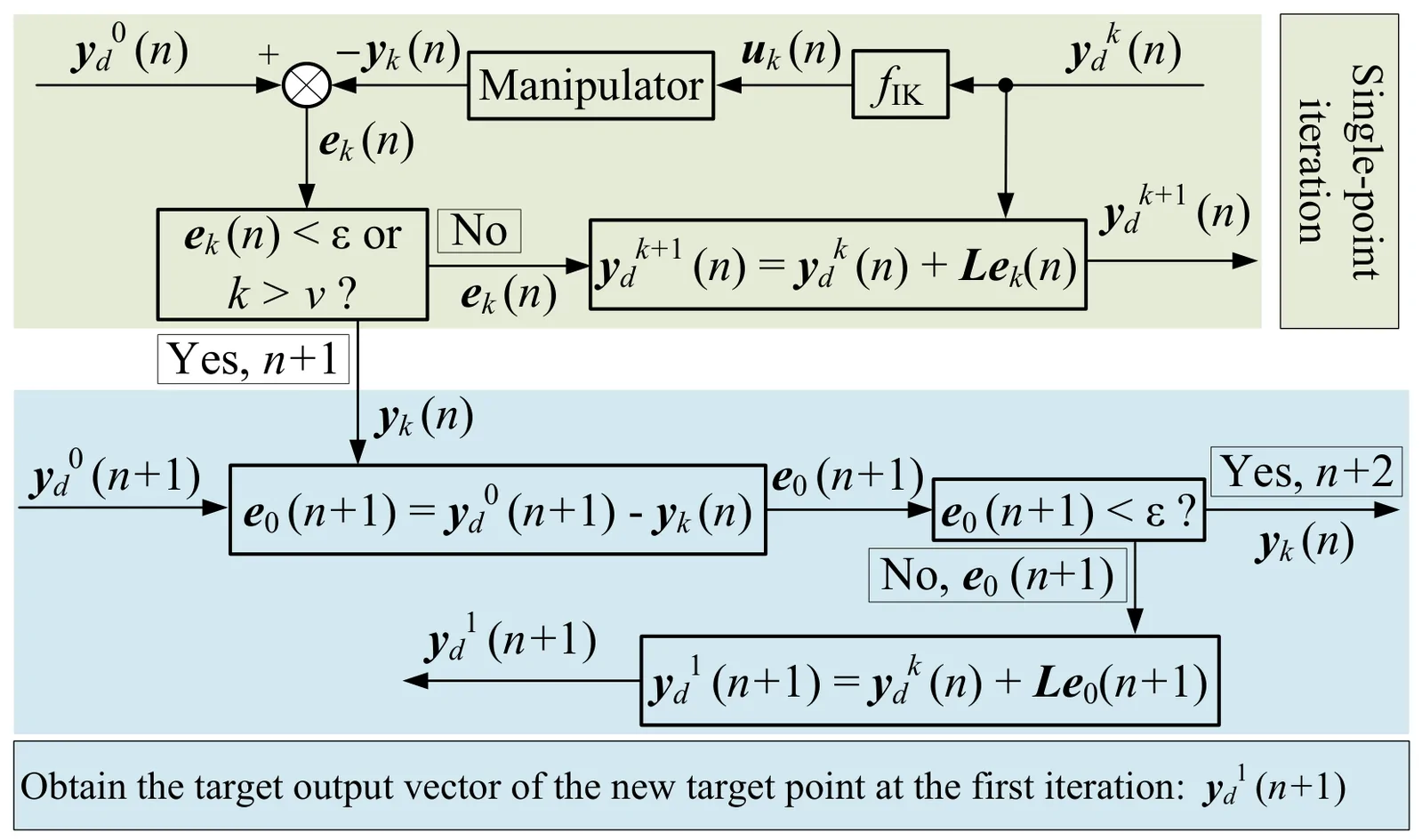
Flowchart of the iterative learning control algorithm for trajectory tracking.

The flowchart of the iterative learning control algorithm for trajectory tracking corresponding to Equation (13) is shown in Figure 10.

#### 3.3.2. Trajectory-Tracking Experiment

To validate the trajectory-tracking performance of the proposed iterative learning control algorithm, a circular trajectory, shown in Figure 6, was selected as the desired trajectory for the soft manipulator. The manipulator was controlled using the iterative learning control algorithm presented in Figure 10. For the first target point, the iteration was terminated when the Euclidean distance error, denoted by σ, satisfied σ<3mm. For the remaining target points, the maximum number of iterations was set to ν=50.

**Figure 11:**
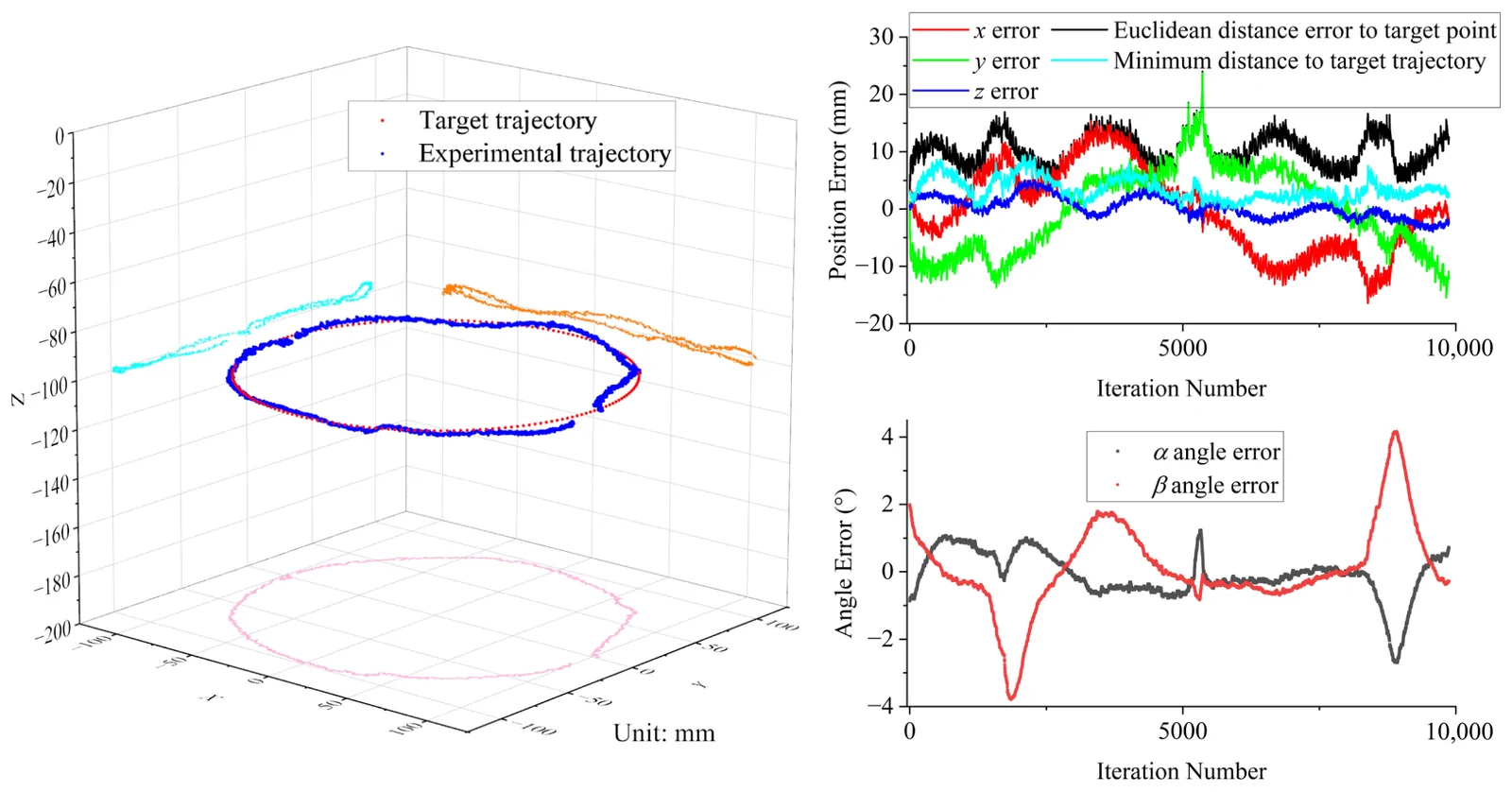
Trajectory-tracking results excluding the first target point.

The trajectory-tracking results are shown in Figure 11, where only the final iteration result of the first target point is presented, rather than its complete iterative process. For the first target point, 339 iterations were required to reduce the Euclidean distance error from 37.57mm to 2.74mm, after which the iterative learning process for the next target point was initiated. The results demonstrate that the proposed algorithm can continuously update the target point and maintain accurate tracking during the trajectory-tracking process. The light-blue curve, labeled as minimum distance to target trajectory, denotes the minimum Euclidean distance from the measured end spatial position to the ideal circular target trajectory, which directly quantifies the trajectory-following deviation.

### 3.4. Performance Comparison with State-of-the-Art Continuum Manipulators

Table 3 summarizes representative tendon-driven and soft continuum manipulators with different control strategies [31,32,33,34]. Direct one-to-one experimental comparison is difficult because these robots adopt distinct mechanical structures, sensor configurations, and test conditions. Nevertheless, this literature-based benchmarking provides a quantitative overview of existing control solutions for continuum manipulators. Benefiting from iterative-learning closed-loop compensation, our scheme can suppress the large hysteresis and nonlinearity inherent to wave-spring soft structures and achieve satisfactory positioning and trajectory-tracking accuracy without precise analytical kinematic modeling.

## 4. Conclusions and Future Work

This paper designs a soft manipulator based on wave spring structures. A model-free inverse kinematics model of the soft manipulator is established via neural network, and an iterative learning control algorithm is further proposed for the manipulator based on the learned model. Experimental validation is conducted to comprehensively evaluate the proposed control scheme. First, point positioning experiments verify the convergence, stability, and generalizability of the designed iterative learning control algorithm. Under high-precision single-point positioning conditions, the control logic of the iterative learning control is tuned and optimized to adapt to continuous trajectory-tracking tasks. Second, circular-trajectory-tracking experiments demonstrate that the developed iterative learning control can achieve high-fidelity spatial trajectory tracking, which verifies the effectiveness and tracking accuracy of the control strategy. Third, target-tracking experiments under external contact disturbances are carried out, and the results prove the strong practicability and disturbance robustness of the iterative learning control method. Distinguished from traditional model-based control schemes that rely on precise analytical kinematics, the proposed framework combines neural network-based inverse kinematics modeling with iterative learning control correction. It can simultaneously realize high-precision single-point positioning and smooth circular trajectory tracking, and maintains favorable tracking performance when the manipulator suffers structural deformation induced by external obstacles. This work provides an effective control solution for wave-spring soft continuum manipulators operating in uncertain environments. Future research will focus on optimizing the iterative update law to accelerate error convergence speed under large transient disturbances, and extend the control framework to more complex spatial trajectories and dynamic collision scenarios.

In terms of execution efficiency, the trajectory-tracking experiment based on the model-free neural network inverse kinematics takes about 75s. By contrast, the full iterative learning control framework consumes approximately 1250s, because multiple iterative learning updates are conducted for each target point along the trajectory, which represents a practical shortcoming of the presented ILC strategy. It should also be noted that the convergence rate of the proposed iterative learning control decreases when the soft manipulator suffers ultra-large deformation, which is caused by aggravated hysteresis and nonlinear friction under large deformation. As a prospective optimization direction, adaptive gain-scheduled ILC will be explored in our future research to alleviate this problem.

In future work, we will further improve the performance of the wave-spring soft manipulator system. First, adaptive gain-scheduled iterative-learning control will be developed to accelerate error convergence under large-magnitude transient external disturbances and mitigate performance degradation under ultra-large deformation. Second, we will extend the proposed control framework to handle more complex three-dimensional spatial trajectories and dynamic collision scenarios. Third, dedicated physical experiments will be carried out to explore the manipulator’s compliant interaction capability, including contact-rich tasks. Fourth, we will generalize the control scheme for multi-segment serial wave-spring manipulators with more degrees of freedom. In addition, computation optimization will be conducted to reduce the runtime overhead of iterative learning updates, paving the way for real-time practical deployment of the wave-spring soft continuum robot.

## Figures and Tables

**Table 1:** Structural geometric parameters of the two-segment wave-spring soft manipulator.

Symbol	S1 (*i* = 1) (unit: mm)	S2 (*i* = 2) (unit: mm)	Parameter Definition
$H_{i}$	$H_{1}=266$	$H_{2}=266$	Total axial length of the *i*-th wave spring segment (including 6 mm planar end caps at both ends)
$L_{pure,i}$	254	254	Pure deformable length of wave spring without end planar sections
*h*	14	14	Vertical wave height between adjacent wave crests and troughs of a single wave unit
*t*	2	2	Wall thickness of wave spring profile strip
$w_{i}$	$w_{1}=6$	$w_{2}=10$	Lateral width of wave spring strip for segment *i*
$d_{i,in}$	$d_{1,in}=41.72$	$d_{2,in}=41.72$	Inner diameter of the *i*-th wave spring skeleton
$d_{i,out}$	$d_{1,out}=53.72$	$d_{2,out}=61.72$	Outer diameter of the *i*-th wave spring skeleton
$r_{hole,i}$	$r_{hole,1}=24$	$r_{hole,2}=28$	Distribution radius of tendon routing holes
$N_{\mathrm{wave}}$	16 pairs (32 wave units)	16 pairs (32 wave units)	Total number of periodic wave units in single segment
$L_{flange}$	15	15	Axial length of connecting flange between S1 and S2
$a_{iu}$	6	6	Vertical height of upper planar end cap
$a_{id}$	6	6	Vertical height of lower planar end cap

**Table 2:** Hyperparameters and training performance of the inverse kinematics neural network.

Parameter	Value
Input dimension	5 (x,y,z,α,β)
Output dimension	6 ($\Delta l_{11}$, $\Delta l_{12}$, $\Delta l_{13}$, $\Delta l_{21}$, $\Delta l_{22}$, $\Delta l_{23}$)
Hidden layer neurons	[256, 128, 64]
Hidden activation	GELU
Output activation	Linear
Dropout rate	0.25
Regularization	L2 ($1\times 10^{-4}$)
Optimizer	Adam
Initial learning rate	0.0003
Loss function	Huber (δ=1.0)
Max training epochs	500
Batch size	64
Test set proportion	0.15
Train set $R^{2}$	0.8135
Test set $R^{2}$	0.8062

**Table 3:** Comparison of control strategies for representative continuum manipulators.

Robot	Algorithm	Performance
6-DoF continuum manipulator [31]	Two machine-learning-based IK solvers	Point tracking (9.67 mm), path tracking (23 mm/37 mm)
OBSS soft manipulator [32]	Real-time vision-based feedback control	Path tracking (13.4 mm)
Echelon 3 manipulator [33]	FEM-based inverse model	Point tracking error (X:4.82mm,Y:55.35mm,Z:7.22mm), path tracking error (X:8.9mm,Y:11.7mm,Z:9.04mm)
TDPCR manipulator [34]	Cosserat-rod kinetostatic model	Point tracking error 4.9mm
Wave-spring manipulator (This work)	NN-based IK + iterative learning control	Point positioning error < 1 mm; circular-trajectory-tracking error < 10 mm

## Data Availability

The datasets generated and analyzed during the current study are available from the corresponding author upon reasonable request.
